# From bench to bedside: the application of cannabidiol in glioma

**DOI:** 10.1186/s12967-024-05477-0

**Published:** 2024-07-11

**Authors:** Shiying Feng, Yuanming Pan, Pu Lu, Na Li, Wei Zhu, Zhiqiang Hao

**Affiliations:** 1 Department of Oncology, Baotou City Central Hospital, Baotou, 014040 China; 2 Central Clinical Medical School, Baotou Medical College, Baotou, 014040 China; 3 Cancer Research Center, Beijing Tuberculosis & Thoracic Tumor Research Institute, Beijing Chest Hospital, Capital Medical University, Beijing, 101149 China; 4 Department of Gynecology, Baotou City Central Hospital, Baotou, 014040 China

**Keywords:** Cannabidiol, Glioma, Preclinical studies, Clinical research, Nanocarriers, Blood–brain barrier

## Abstract

Glioma is the most common malignant tumor in central nervous system, with significant health burdens to patients. Due to the intrinsic characteristics of glioma and the lack of breakthroughs in treatment modalities, the prognosis for most patients remains poor. This results in a heavy psychological and financial load worldwide. In recent years, cannabidiol (CBD) has garnered widespread attention and research due to its anti-tumoral, anti-inflammatory, and neuroprotective properties. This review comprehensively summarizes the preclinical and clinical research on the use of CBD in glioma therapy, as well as the current status of nanomedicine formulations of CBD, and discusses the potential and challenges of CBD in glioma therapy in the future.

## Introduction

Glioma is one of the most prevalent primary malignant tumors in the central nervous system (CNS). According to World Health Organization (WHO) guidelines, glioma is categorized into grades I through IV based on histological criteria, with the malignancy level escalating with each grade. Glioblastoma Multiforme (GBM), a grade IV astrocytoma, is the most aggressive form, accounting for approximately 48.6% of malignant tumors in the CNS and about 70% of all gliomas [1, 2]. The standard treatment regimen for adult GBM patients involves post-surgical radiotherapy in conjunction with temozolomide (TMZ) chemotherapy. However, due to a low percentage of TMZ-responsive individuals (< 50%) [3], coupled with the tumor’s highly invasive nature, hypoxia tolerance, immune evasion, and the difficulties associated with complete surgical resection [4], the median survival period post-treatment for GBM patients is between 12 and 15 months, with a five-year relative survival rate of about 5% [1, 5]. The median survival period drops to between 1 and 10.8 months for patients with recurrent GBM, and no standard treatment protocols currently exist [6]. Importantly, normal neuroglial cells exhibit a considerable degree of radio-resistance, whereas adult neurons and endothelial cells are highly sensitive to ionizing radiation, whether used alone or in combination with chemotherapy. Cranial irradiation can lead to severe cognitive deficits due to damage to neurons and endothelial cells. Thus, the treatment of gliomas must not only be effective but also safer.

Cannabidiol (CBD), is second to tetrahydrocannabinol (THC) in most varieties of cannabis and is known for its non-psychoactive and non-addictive properties [7]. Studies both in vitro and in vivo have exhibited that it can inhibit the malignant proliferation of various solid tumors (including glioma, breast cancer, and prostate cancer) [8, 9]. The anticancer mechanisms of glioma are multifaceted, involving cell cycle arrest, inhibition of proliferation, induction of autophagy and apoptosis, and suppression of cell adhesion, migration, angiogenesis, and metastasis [10–13]. Additionally, research indicates that CBD can synergize with TMZ, reversing glioma cells’ resistance to TMZ; it can also sensitize tumors to radiotherapy. Moreover, due to its anti-inflammatory and neuroprotective effects, CBD can alleviate side effects associated with tumor growth and antitumor treatments, such as pain, epilepsy, nausea, vomiting, and neuroinflammation [14–16]. Faced with the grim prognosis for glioma patients and the myriad limitations of current treatment modalities, the demand for innovative therapeutic approaches is urgent. Despite the challenges posed by CBD’s high lipophilicity in the design of drug delivery systems, overcoming these hurdles through nanotechnology (e.g., liposomes, microemulsions) can enhance drug loading, bioavailability, and therapeutic efficacy.

This review aims to explore the roles and clinical values of CBD as a promising therapeutic candidate in the preclinical and clinical trials for glioma therapy. It will also review the current status of CBD nanoparticle formulations designed for improved bioavailability and biocompatibility, and for targeting gliomas across the blood–brain barrier (BBB). This discussion not only illuminates the therapeutic prospects of CBD in glioma treatment but also addresses the current issues and challenges in its applications, providing readers with a comprehensive and objective understanding of CBD.

## Preclinical studies of CBD in glioma

**Fig. 1:**
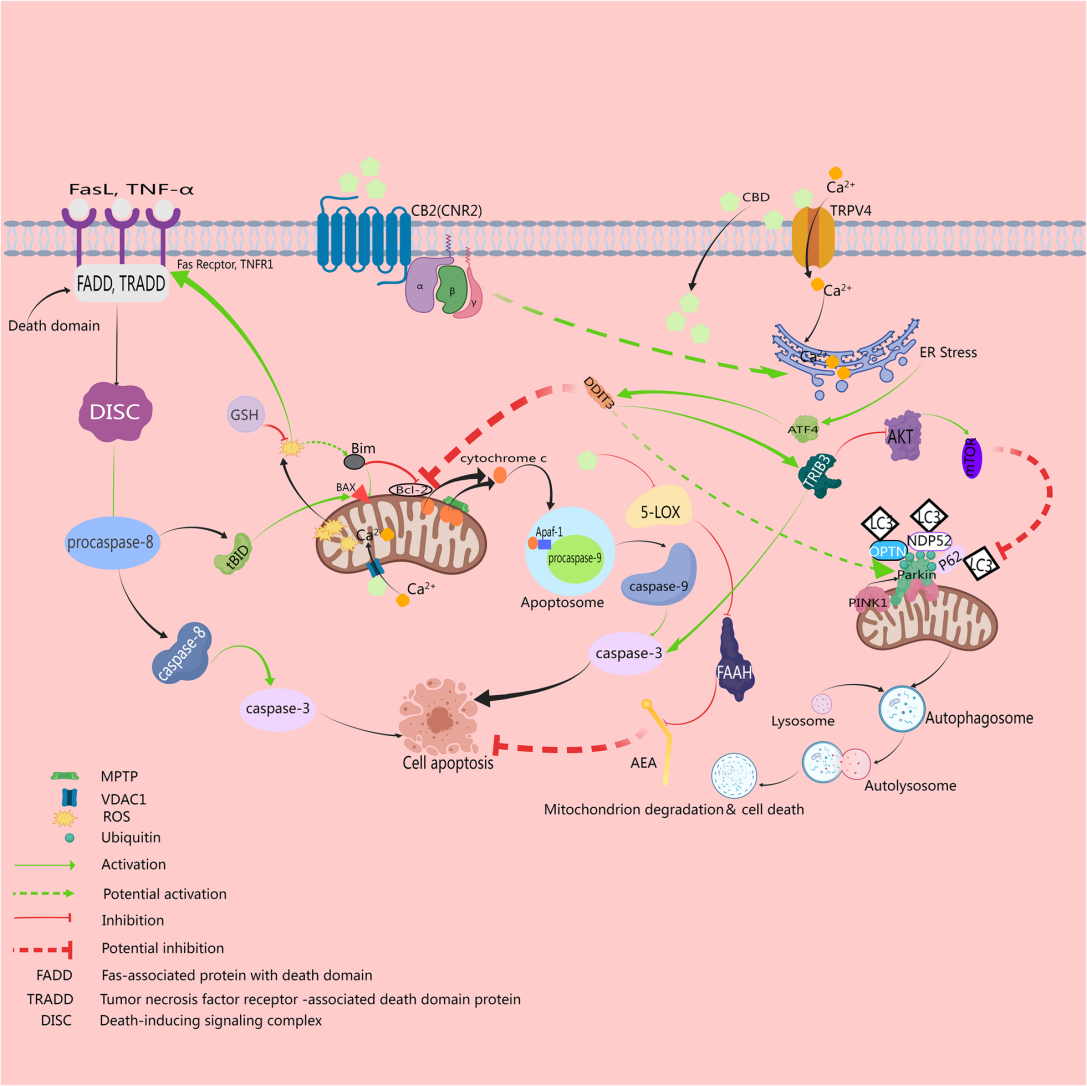
CBD directly exhibits anti-glioma effects. CBD chiefly induces glioma cellular apoptosis via both intrinsic pathways (mitochondria) and extrinsic pathways (death receptor), wherein cannabidiol, through oxidative stress and the activation of multiple receptors such as TRPV4, VDAC1, and endogenous cannabinoid receptors, alters intracellular calcium flux and AEA levels. These alterations compromise mitochondrial and endoplasmic reticulum functionality, subsequently triggering apoptotic and autophagic downstream cascades and intertwining the two, ultimately curtailing glioma cell proliferation and precipitating their demise. It is evident that the disruption of mitochondrial structures/functions and the elicited ER stress are the central of CBD's anti-glioma efficacy. This figure is created with MedPeer (medpeer.cn)

**Fig. 2:**
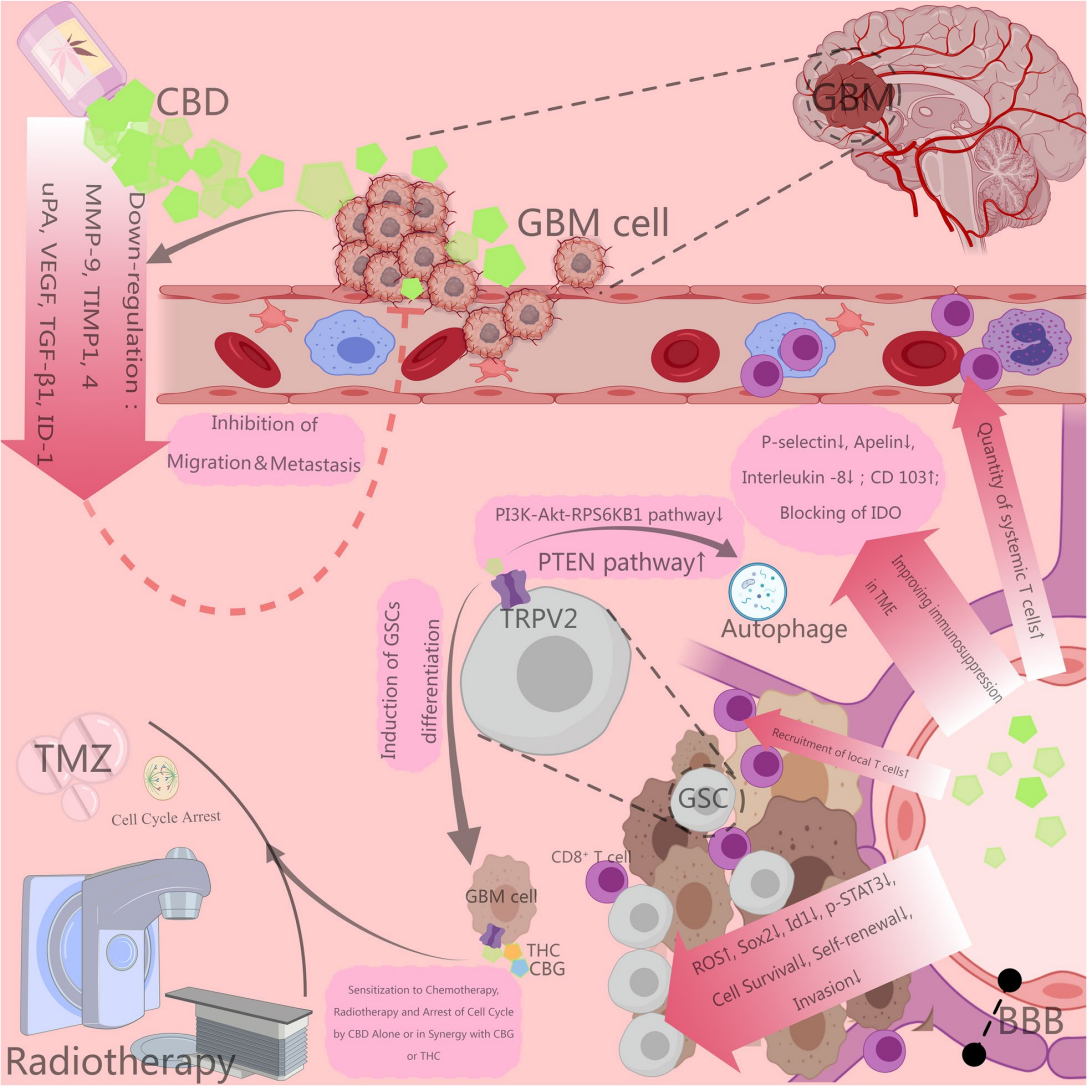
CBD indirectly exhibits anti-glioma effects. CBD impedes glioma cellular migration and invasion by downregulating the expression of proteins such as MMP-9, TIMP1,4 and VEGF; CBD enhances the recruitment of cytotoxic T cells systemically and locally by upregulating CD103, obstructing the immunological checkpoint IDO, and reducing the expression of p-selectin, thereby ameliorating the immunosuppressive state of GBM tumor microenvironment. Furthermore, through its interaction with TRPV2, it mediates the inhibition of the PI3K-Akt-RPS6KB1 pathway and activation of PTEN pathway, inducing autophagy and differentiation in GSCs. CBD, either used singularly or in conjunction with THC or CBG, can arrest cell cycle in glioma cells and potentiate the efficacy of radiochemotherapy. This figure is created with MedPeer (medpeer.cn)

In vitro and animal model studies have demonstrated that CBD can influence the survival of cancer cells by activating multiple targets and signaling pathways. The mechanisms of CBD’s anticancer effects may involve promoting apoptosis, increasing autophagy, inhibiting the proliferation and migration of tumor cells, counteracting tumor angiogenesis [17], and enhancing the sensitivity of tumor cells to radiotherapy and chemotherapy [18]. The actions and mechanisms of CBD mentioned in glioma-related preclinical studies include the following (see Figs. 1 and 2 for detailed graphical representation):

### CBD exhibits the anti-proliferation effect in glioma

The pioneering study by Jacobsson et al. in 2000 unveiled that CBD demonstrated a notable anti-tumor effect after six days of co-cultivation with the rat C6 glioma cell line [19]. This phenomenon was further substantiated by Massi and colleagues in 2004, revealing that CBD, at an IC_50_ of 25 μM, inhibited the proliferation of glioma cells (U-87MG and U-373MG) in a time- and dose-dependent manner by inducing apoptosis [20]. For the first time, the authors proposed that CBD induces apoptosis by activating the CB2 receptor, triggering cellular oxidative stress-manifested by the generation of reactive oxygen species (ROS) and depletion of glutathione, thereby activating caspase-8, caspase-9, and caspase-3 to induce cell death [21]. Recent studies have identified an upregulation of CB1, CB2, and transient receptor potential vanilloid 2 (TRPV2) receptors in gliomas, suggesting that phytocannabinoids such as CBD may exert antitumor effects through interactions with these receptors [22]. Previous research has demonstrated that, in vivo, THC can inhibit cancer cell proliferation and invasion, and induce apoptosis by activating CB1 and CB2 receptors [23]. Although CBD has a relatively low affinity for cannabinoid receptors, its potential to exert antitumor effects via CB1 and CB2 receptor interactions warrants further investigation [24]. Further investigations using animal models and cellular experiments revealed that CBD's antiproliferative action might also rely on the lipoxygenase (LOX) pathway. Specifically, CBD treatment suppressed the expression of 5-LOX and increased the production of fatty acid amide hydrolase (FAAH), which led to a reduction in anandamide (AEA) synthesis, ultimately inhibiting the viability of glioma cells [25]. Additionally, CBD-treated glioma cells exhibited oxidative stress, typically evidenced by increased ROS production. To counteract this damage, cells upregulated a plethora of heat shock protein (HSP) superfamily genes, whose increased expression diminished the cytotoxic effects of CBD. The introduction of HSP protein inhibitors could restore the cytotoxic efficacy of CBD [26].

### CBD facilitates the induction of apoptosis and autophagy through endoplasmic reticulum stress and mitochondrial damage in glioma

Mitochondria, the cellular powerhouses responsible for ATP production, play a pivotal role in cell cycle regulation, cell death, and signal transduction. In cancer cells, alterations in mitochondrial function and dynamics are closely associated with tumor growth, metabolic reprogramming, drug resistance formation, and immune regulation. Furthermore, endoplasmic reticulum (ER), the critical site for protein folding and post-translational modifications, its dysfunction (ER stress) is linked to the onset and progression of tumors. ER stress, an important target for antitumor drugs, its activation can induce cell autophagy and apoptosis, which are important for maintaining intracellular homeostasis and regulating tumor cell survival and death.

Gross and colleagues discovered that CBD induces apoptosis in glioma cells mediated by caspase activation and triggers mitochondrial dysfunction mediated by cellular toxicity through activating the voltage-dependent anion channel 1 (VDAC1) on the outer mitochondrial membrane, leading to mitochondrial calcium homeostasis imbalance [27]. Huang and others found that CBD activates the TRPV4, causing extracellular calcium influx that induces ER stress and mitochondrial autophagy in glioma cells through ATF4-DDIT3-TRIB3-AKT-mTOR axis, ultimately leading to lethal autophagic cell death [28]. Rupprecht and team demonstrated that CBD + THC inhibited glioma cellular energy metabolism and exerts the anti-tumor effect by affecting the mitochondrial electron transport chain through inhibition of subunits in mitochondrial complexes I and IV, impacting mitochondrial respiration [29]. Moreover, CBD induces ER stress by triggering cellular oxidative stress and causing calcium ion imbalance within the endoplasmic reticulum, thereby inhibiting glioma cellular proliferation and inducing apoptosis [30]. Collectively, it can be inferred that the structural and functional impairments of mitochondria induced by CBD, such as changes in mitochondrial energy metabolism, occupy an important value in its anti-tumor mechanism; the pathways of cell apoptosis and autophagy on which CBD's anti-glioma activity depends are not isolated but interconnected.

### CBD inhibits the migration, invasion, and angiogenesis of glioma cells

Gliomas, particularly GBM, pose a grave threat to patient survival. These tumors are notorious for their rapid proliferation and their propensity to invade neighboring brain regions, rendering treatment exceedingly challenging. Thus, delving into and deciphering the mechanisms and strategies that can curb the migration, invasion, and angiogenesis of these tumor cells is of paramount importance. In this realm, the research on CBD is particularly promising.

Studies conducted by Vaccani et al. using Boyden chamber assays have revealed that CBD, in concentrations ranging from 0.01 to 9 μM, inhibits the migration of the U-87MG glioma cell line without relying on the endogenous cannabinoid receptor mechanism [31]. Furthermore, investigations by Solinas et al. have shown that even at low concentrations (1–12 μM), CBD significantly curtails the invasiveness of GBM. CBD treatment results in the downregulation of key proteins associated with tumor invasion and angiogenesis, such as matrix metalloproteinase-9 (MMP-9), tissue inhibitors of metalloproteinase 1 and 4 (TIMP1/4), urokinase plasminogen activator (uPA), SerpinE1-plasminogen activator inhibitor type-1 (Ser-PAI-1), vascular endothelial growth factor (VEGF), transforming growth factor-β1 (TGF-β1), C-X-C motif chemokine 16 (CXCL-16), platelet-derived growth factor-AA (PDGF-AA), monocyte chemotactic protein-1 (MCP-1), angiogenin, and hypoxia-inducible factor-1α (HIF-1α) [32]. Additionally, the deoxyribonucleic acid (DNA)-binding inhibitor ID-1, which is associated with glioma invasiveness, is also inhibited by CBD [33, 34]. The regulation of these molecules by CBD reflects its capacity to inhibit tumor progression through a multi-targeted effect.

### CBD exerts anti-glioma effects by modulating systemic immunity in tumor microenvironment

The therapeutic approaches for glioma are currently at an impasse, stagnant and unprogressive. Although immunotherapy, as a burgeoning treatment modality, has shown the remarkable promise. GBM, characterized as an immunologically "cold" tumor, demonstrates inherent systemic and localized immunosuppression, particularly evident in the scarcity of tumor-infiltrating T cells. This characteristic significantly diminishes the efficacy of existing immunotherapeutic interventions for GBM [35]. However, recent scientific advancements have shed light on CBD's potential role in modulating systemic immune responses and the tumor microenvironment, offering novel insights into surmounting this challenge.

Zhou and colleagues’ research elucidates that CBD may enhance the immunological landscape of GBM through the stimulation of T-cell proliferation and the activation of antigen-presenting cells [36]. Moreover, CBD's ability to augment the expression of CD103 and antigen presentation further amplifies the immune response of CD8 + T cells. By inhibiting P-selectin, apelin, and interleukin-8, as well as obstructing the immune checkpoint indoleamine 2,3-dioxygenase (IDO), CBD is posited to alter tumor microenvironment dynamics within mice bearing intracranial GBM, heralding a significant stride towards immunotherapeutic breakthroughs in glioma therapy [37].

### CBD inhibits glioma stem cells (GSCs) viability and stem-like characteristic expression

GSCs, owing to their distinctive properties such as their capacity for self-renewal, their role in reshaping the tumor microenvironment, their mechanisms of resistance to treatment, and their contributions to tumor angiogenesis and cellular heterogeneity, occupy a pivotal position in initiation, progression, recurrence, and drug resistance of glioma [38, 39]. The ability of GSCs to perpetually divide and generate novel tumor cells underpins and propels tumor growth. These cells exhibit a profound resistance to current treatment modalities, including radiotherapy and chemotherapy, largely attributable to their enhanced DNA repair capabilities, representing a key obstacle to the successful treatment of glioma and a significant factor in treatment failure and disease recurrence. Therefore, the development of targeted therapeutic strategies against GSCs is of paramount importance in the battle against glioma [40]. Such strategies might encompass the development of drugs that can specifically eliminate GSCs or inhibit their function, the utilization of immunotherapeutic approaches to activate immune responses against GSCs, or the development of treatments that disrupt the interactions between GSCs and their microenvironment. Recent research into the effects of CBD on GSCs has undoubtedly illuminated a path toward the conclusion of this battle.

Investigations by Singer and colleagues into primary GSCs have demonstrated that CBD can inhibit the vitality and stem-like characteristics of GSCs by inducing the generation of ROS, activating the p38 pathway, and downregulating Sox2, Id1 and p-STAT3 [41]. Additionally, CBD can activate TRPV2 and induce autophagy in GSCs through the PI3K-AKT-RPS6KB1/PTEN pathway, as well as promote GSCs differentiation by upregulating Aml-1a. Given that GSCs retain the ability to respond to physiological signals that induce the differentiation of neural stem cells (NSCs) into neurons, astrocytes, and oligodendrocytes, mechanism-induced differentiation may present a promising strategy for eradicating this tumor-driving cell population. Activation of TRPV2 channels can negatively regulate glioma cell survival and proliferation, while also promoting the differentiation of glioma stem cells. These actions collectively result in the inhibition of GSC proliferation [42]. CBD also facilitates the DNA binding of the glioma cell NF-κB subunit RELA, while preventing the phosphorylation of RELA on Serine-311 through the downregulation of protein kinase C ζ (PKCζ), with the sustained DNA binding of non-phosphorylated Serine-311 RELA mediating GSC cytotoxicity. Remarkably, in CBD-sensitive GSCs, the in vitro concentration and time course of CBD outperformed the chemotherapeutic drug TMZ. Furthermore, widespread sensitivity to CBD was observed in GSC cohorts with low levels of ROS, whereas high ROS content inhibited CBD-induced GSCs’ death, which proposed that ROS level could serve as the predictive biomarker for CBD-sensitive tumors and that the combined administration of a BBB-permeable ROS scavenger (such as the anti-hypertension drug captopril, acting as a thiol donor) might enhance the therapeutic efficacy of CBD [43].

### CBD alone or in synergy with THC/CBG sensitizes chemo- and radiotherapies and enhances anti-glioma effects

Chemotherapy and radiotherapy hold critical positions in the treatment of gliomas. However, the gradual development of resistance by tumor cells to these treatments poses a significant challenge in the management of gliomas [44, 45]. CBD also presents a potential solution to this issue.

Nabissi and colleagues found that CBD enhanced the uptake and sensitivity of cells to chemotherapeutic drugs (such as doxorubicin) by increasing TRPV2 expression and activating the TRPV2 channel [46]. Research by Torres et al. indicates that a combination of CBD, THC, and TMZ exhibits a synergistic effect, inducing autophagy-mediated apoptosis in glioma cell lines (such as U-87MG and T98G) and overcoming TMZ resistance in orthotopic xenografts of glioma in nude mice [47]. In vivo, the synergistic anti-glioma action of CBD and THC was further confirmed in a subcutaneous tumor xenograft model in athymic nude mice using U-87MG cells, demonstrated by anti-tumor cell proliferation (identified by Ki-67), induction of apoptosis (identified by TUNEL), and anti-tumor angiogenesis (identified by CD31 immunostaining) [48].

Neurons, endothelial cells, and NSCs exhibit high sensitivity to ionizing radiation, whether applied alone or in combination with chemotherapy. Clinical observations and animal studies have demonstrated that cranial irradiation can lead to severe cognitive deficits due to damage to neurons and endothelial cells, as well as inhibition of NSC proliferation and induction of cell death [49, 50]. Scott and colleagues discovered that pre-treating various glioma cell lines (T98G, U-87MG and GL261) with THC and CBD for 4 h increased their radiosensitivity, a change associated with increased autophagy (indicated by elevated LC3B-II) and apoptosis [51]. This enhancement in radiosensitivity was also correlated with the upregulation of p-JNK1/2 and MAPK p-p38 levels and the downregulation of p-ERK1/2 and p-AKT1 levels [52].

Marcu et al. demonstrated that CBD and THC have a synergistic effect in vitro on glioblastoma cell lines (U-87MG, U251 and SF126), manifested by inhibiting glioma cellular proliferation (through downregulating pERK), activating apoptosis induced by CB2 receptor activation and ROS generation, and blocking the cell cycle [23]. Despite the promising outcomes of combining CBD with THC against gliomas, the psychoactive properties of THC necessitate consideration of its psychiatric side effects and potential for addiction as a medication. Seeking an alternative to THC, Lah and colleagues discovered that cannabigerol (CBG) exhibits broad anti-cancer activity in glioblastoma. The combination of CBD and CBG was more effective than CBD with THC, inducing caspase-dependent apoptosis and inhibiting glioma cellular invasiveness [53, 54].

These studies indicate that the combination of CBD with THC/CBG can produce a synergistic effect against glioma and sensitize to radiotherapy and chemotherapy. This has significant implications for glioma treatment: maintaining the same or even better therapeutic effects while reducing treatment doses could minimize toxic side effects and improve treatment tolerance.

### Emerging mechanisms and applications of CBD in glioma treatment

In the realm of glioma treatment research, the application of cannabidiol continues to expand, encompassing a range of innovative mechanisms and strategies. Recent studies have unveiled several emerging mechanisms of action, which further enhance the potential of CBD in the treatment of glioma.

One such emerging mechanism involves the interaction of CBD with stress granules (SGs), a type of biomolecular condensate (BMC), which plays a crucial role in cellular stress responses and has been implicated in anti-glioma activity. SGs, also known as ribonucleic acid (RNA)-protein complexes, were first described as dense cytoplasmic granules appearing in mammalian cells subjected to heat shock [55, 56]. SGs influence mRNA translation and stability, and are associated with apoptosis, signal transduction, and gene regulation; additionally, they are closely linked to tumorigenesis, tumor progression, and drug resistance [57, 58]. Recent pioneering research by Wang et al. demonstrated that CBD treatment significantly upregulates SGs in GBM, and bioinformatic analyses suggest that CBD may regulate the formation and increase of SGs in GBM through related receptors and genes, as well as induce translational stalling, thereby exerting its anti-glioma effects; furthermore, some research indicates that SGs can affect tumor angiogenesis and stemness expression during cancer progression, areas where previous research has confirmed CBD's efficacy [59]. Thus, the authors propose that SGs could be a potential therapeutic target for GBM. It is reasonable to hypothesize that the dynamic changes in SG formation within GBM may serve as a mediator and concrete manifestation of CBD's complex, multi-pathway anti-glioma functions.

Additionally, CBD has been found to induce ferroptosis in GBM through ROS and p-ERK pathways, a regulated cell death mechanism driven by iron-dependent lipid peroxidation, thus providing a novel therapeutic avenue for glioma treatment [60].

These innovative strategies highlight the expanding therapeutic potential of CBD and underscore the importance of continued research to fully elucidate its mechanisms and optimize its clinical application in glioma management.

### Debates and controversies

Prior discussions highlighted the potential of CBD in combating gliomas through various mechanisms, such as anti-proliferation and anti-invasion, with its antitumor effects seemingly sparing normal neuronal cells [61, 62]. However, the specific molecular underpinnings of these processes remain partially obscure, fueling ongoing debates.

For instance, it has been discovered that, when CBD is used in conjunction with chemotherapy agents, a notable issue arises: its cytotoxic effects on tumor cells lack specificity, potentially posing a risk to the CNS. Moreover, the synergy between CBD and chemotherapy manifests within a remarkably narrow dosage window, with antagonistic effects potentially occurring at dominant concentration levels [63].

Another contention revolves around whether CBD primarily induces apoptosis or autophagy in glioma cells, leading to cytotoxicity. The role of autophagy in glioma cells-as either a protective or toxic mechanism-varies, with researchers holding differing perspectives, and a consensus remains elusive. Autophagy has long been regarded as a double-edged sword in cell survival and death. On one hand, autophagy enhances cell survival by removing damaged intracellular components, thereby improving the cell's ability to withstand various stresses, and, in cancer cells, autophagy may facilitate survival during various treatments [64, 65]. Therefore, some studies have suggested that inhibiting autophagy can enhance the sensitivity of tumor cells to chemotherapeutic agents [66]. On the other hand, upregulation of autophagy can lead to the degradation of essential organelles, thereby inducing apoptotic cell death [67, 68]. This is a process of quantitative change leading to qualitative change. It is also an interwoven process. There is crosstalk between autophagy and apoptosis: autophagy can inhibit the induction of apoptosis, while apoptosis can suppress the protective autophagic process. Interestingly, at the molecular level, we also observe crosstalk between autophagy and apoptosis. Beclin 1, a key protein promoting autophagy, exhibits anti-apoptotic effects in various contexts. Conversely, caspases, which are essential proteins in apoptosis, can cleave Beclin 1 during apoptosis, thereby impairing its autophagic function [69, 70]. Additionally, autophagy can directly induce the death of glioma cells through autophagy cell death (ACD) [71]. The fate of cells varies depending on the specific intervention points of drug-induced autophagic flux.

CBD's actions may encompass both the promotion of apoptosis and the induction of autophagy, with the specific mechanisms likely dependent on cell type (cellular heterogeneity), influenced by drug dosage and duration of exposure, and possibly regulated by the interplay of intracellular signaling pathways, or more so, the crosstalk between CBD-induced autophagy and apoptosis pathways. Hence, the scientific discourse surrounding these functions of CBD and how they synergize to induce death in glioma cells persists. To unravel the enigma of these actions, further studies are imperative to elucidate these complex processes. Additionally, considering the discrepancy between cellular models and solid tumors, the models may not fully replicate the intricate microenvironment and biological behaviors of solid tumors, necessitating further precisely designed randomized controlled trials to optimize therapeutic strategies for specific glioma types and unique backgrounds.

## Clinical investigations of CBD in the treatment of glioma

While numerous preclinical studies have delineated the anticancer effects and mechanisms of CBD on gliomas, clinical trials in this realm remain scarce (see Table 1). This section provides a comprehensive overview of CBD's clinical application in the treatment of glioma, focusing on two main aspects: 1. Clinical trials of CBD for glioma therapy; 2. Research on the use of CBD in managing glioma-related symptoms and treatment-associated symptoms.

### Clinical trials of CBD for glioma treatment

GW Pharmaceuticals conducted a pivotal phase Ib clinical trial (NCT01812603, NCT01812616) in 2014, aiming to assess the safety, tolerability, and efficacy of a sublingual spray, Sativex^®^ (containing 27 mg/ml THC and 25 mg/ml CBD), in combination with dose-intense temozolomide (DIT) therapy in patients with recurrent GBM [6]. The trial comprised two parts: the initial segment was a non-randomized, open-label design intended to evaluate the safety and side effects of combining Sativex^®^ with TMZ, involving six patients with grade IV GBM who had undergone radiotherapy and TMZ treatment after their first recurrence. Sativex^®^ was administered through gradual dose escalation to the maximum tolerated dose (100 μl per spray, up to 12 sprays/day) applied to the oral mucosa in conjunction with TMZ treatment. Results indicated that 50% of the patients discontinued treatment due to adverse events, with the most common adverse events being mild to moderate fatigue, dizziness, headache, and vomiting. The subsequent randomized, double-blind, placebo-controlled portion included 21 patients, divided into an experimental group receiving Sativex^®^ + TMZ and a control group receiving placebo + TMZ. Findings showed that the 1-year survival rate in the Sativex^®^ group (83.3%) was significantly higher than that in the placebo group (44.4%, p = 0.042), albeit with a higher severity of adverse events. Sativex^®^ did not affect the pharmacokinetics of TMZ, suggesting that combined administration is feasible. Overall, the trial demonstrated that the personalized dosing regimen of Sativex^®^ in combination with DIT has good safety and tolerability in patients with recurrent GBM, potentially contributing to an increase in survival rates (the first part achieved a 6-month progression-free survival (PFS6) of 16.7% and a 1-year survival rate of 50.0%; the second part had a PFS6 of 33.3% and a 1-year survival rate of 83.3%). This contrasts sharply with the previously reported poorer PFS6 (9%) and 1-year survival rate (14%) in patients with recurrent GBM [72]. However, despite these conceptually revolutionary results, limitations of the trial include a small sample size and the potential for confounding factors between different cohorts that could affect outcomes. Future clinical trials are needed to replicate these findings in a larger patient population and further investigate the mechanisms and optimal treatment protocols.

In the pursuit of understanding the potential applications of CBD in various cancer treatments, Julian et al. embarked on a four-year open-label clinical trial [73]. This study engaged 119 cancer patients, encompassing a spectrum of cancers such as prostate, breast, esophageal, and gliomas. Within this trial, participants received synthetic pharmaceutical-grade CBD oil droplets provided by STI Pharmaceuticals, with each droplet containing 1 mg of CBD and a total concentration of 5% (w/v). The administration followed a cyclic regimen: three days of dosing followed by a three-day hiatus, with an average dosage of 10 mg per administration, twice daily, and, if necessary, escalated to a maximum of 30 mg per dose. The research findings indicate that a significant therapeutic effect of CBD necessitates no less than a six-month treatment duration, during which no notable adverse effects were observed. Particularly for glioma patients, out of ten individuals, seven were diagnosed with GBM; the authors noted that four exhibited an extension in median survival time, while three showed a trend towards slowed tumor progression. For patients with anaplastic ependymoma, three participants in the trial also experienced an extended median survival time. Additionally, unique case reports highlighted the individual responses of a five-year-old boy and a fifty-year-old patient, both of whom demonstrated improvements after transitioning to CBD treatment following other therapeutic interventions. Nevertheless, the study is marked by significant limitations: The absence of a randomized control group undermines the objectivity and accuracy of CBD's efficacy; case data lacks comprehensive quantitative analysis, and the clarity and transparency of dosage adjustments and administration standards are insufficient; Moreover, the relatively small sample size and the lack of thorough analysis of heterogeneity among different cancer types constrain the generalizability of the conclusions.

Several clinical trials concerning the treatment of gliomas with CBD are currently underway, including: the Spanish Neuro-Oncology Group (GEINO) is conducting the GEINO-1601 trial (NCT03529448); a Phase I clinical trial (NCT03246113); the ARISTOCRAT II (NCT05629702); a study conducted by Leaf Vertical Inc (NCT03607643). (see Table 1 for more details).

### Research on CBD in managing glioma-related symptoms and treatment-associated symptoms

#### Research on CBD in patients with glioma associated epilepsy

Seizures are indeed a common complication in patients with brain tumors, with their incidence varying based on the location and biological characteristics of the lesion [74]. Patients with low-grade glioma (WHO Grade I–II) tend to have a slightly higher incidence of epilepsy than those with high-grade glioma (WHO Grade III–IV). Specifically, 60–75% of patients with low-grade glioma experience seizures, especially when the lesions located in superficial cortical areas or the insular region. In contrast, the incidence ranges from 25 to 60% in higher-grade glioma, with about 40–45% of patients with GBM experiencing seizures. The emergence of drug-resistant epilepsy also poses a significant challenge [75–77]. The treatment of epilepsy in glioma patients is challenging. The most common approaches include surgery, radiotherapy, and conventional antiepileptic drugs. However, each of these treatments has limitations. Surgery might not be feasible for tumors located in functional areas or critical brain regions, and post-surgery, 20–35% of patients may not achieve effective seizure control [78]. Radiotherapy can cause neuronal damage leading to cognitive impairments, and its control rate is relatively low [79]. Traditional antiepileptic drugs can have issues with drug resistance and interactions, especially when using CYP3A4 enzyme inducers (such as carbamazepine, phenytoin, and phenobarbital), which need to be considered alongside chemotherapy medications, and seizures and the use of antiepileptic drugs can lead to cognitive decline and reduced quality of life [80–82]. Therefore, finding a safe, tolerable, and effective medication to control seizures in glioma patients is a pressing need for both clinicians and patients.

Researchers from the University of Alabama at Birmingham have focused on exploring the safety and efficacy of prescription-level CBD (specifically Epidiolex^®^) in patients with tumor-related epilepsy enrolled in their Birmingham CBD program (NCT02700412 for children and NCT02695537 for adults). In this study, three patients with refractory seizures due to primary brain tumors received escalating doses of CBD, starting at 5 mg/kg per day in two divided doses, increasing at a rate of 5 mg/kg every two weeks to a maximum of 50 mg/kg per day. The frequency and severity of seizures during the follow-up period were reported using monthly average seizure counts and the Chalfont Seizure Severity Scale (CSSS). Findings indicated that two of the observed patients showed a reduction in seizure frequency, and all three exhibited improvements in seizure severity. Additionally, a linear relationship between CBD dosage and plasma levels was observed, suggesting that higher CBD doses/levels are associated with a higher response rate (RR) in seizure improvement. These preliminary findings support further research into the potential of CBD as a treatment for epilepsy associated with brain tumors [83, 84].

#### The role of CBD in alleviating pain and anxiety symptoms in glioma patients and its impact on quality of life

GBM patients indeed face significant challenges beyond the primary illness, including symptoms of anxiety, depression, pain, and sleep disturbances, all of which can considerably diminish their quality of life. Pain associated with cancer, experienced by 55.0–66.4% of patients during treatment and in advanced stages of the disease, poses a significant hurdle [85]. Despite adherence to the WHO's three-step ladder for cancer pain relief, a subset of patients continue to suffer from breakthrough and chronic pain that is inadequately managed by opioids (10–15% of patients do not achieve sufficient relief), and opioid use sometimes leads to severe adverse effects. The quest for novel analgesics remains critical due to the limitations of current pain management strategies and the detrimental impact of pain on patients' functionality and mental health. In this context, CBD has garnered attention as a potential symptom management option [86–89].

Recent clinical studies have evaluated the efficacy of the cannabinoid-based medicine Sativex^®^ in alleviating persistent chronic pain in patients with advanced cancer. Two pivotal Phase III trials by Lichtman et al., registered as NCT01361607 and NCT01424566, used double-blind, randomized, placebo-controlled designs but differed in methodology. The first study employed a conventional randomized clinical trial design, while the second utilized an enriched enrollment, randomized withdrawal design aimed at identifying a subset of patients who respond well to active treatment. Although Sativex^®^ did not achieve significant results in the primary endpoints (percentage improvement in mean daily pain Numeric Rating Scale (NRS) score in study 1 and mean change in pain NRS score from baseline to the end of treatment in study 2), it showed benefits in several secondary endpoints, such as improvements in mean pain scores and sleep disruption scores, particularly among patients under 65 years old in the United States (p = 0.040) [90, 91]. Another significant study highlighted the advantages of using Sativex^®^ over THC alone in managing intractable cancer-related pain. This multicenter, double-blind, randomized, placebo-controlled clinical trial demonstrated that Sativex^®^ was more effective in reducing pain by at least 30% compared to placebo (p = 0.006), while THC alone had a similar effect to placebo [89]. Further research confirmed the good tolerability and sustained analgesic effects of long-term use of Sativex^®^ [92]. A "N of 1" randomized, double-blind, placebo-controlled crossover trial reported by William et al. also supported the benefits of combined CBD and THC administration, which was significantly more effective in alleviating chronic, neuropathic pain symptoms compared to THC alone [93]. Similar findings were validated in a trial by Portenoy et al., further affirming the positive effects of Sativex^®^ in adjunctively treating chronic pain in advanced cancer patients unresponsive to opioids [94]. Collectively, these studies underscore the potential of cannabinoid-based medications in supplementing the relief of persistent chronic pain caused by cancer, especially where traditional opioid analgesics fail to provide adequate relief.

In the exploration of CBD's potential applications in pain management, particularly its efficacy in intervening in chronic pain and pain associated with cancer as an unconventional therapy, it is equally imperative to scrutinize the role of CBD in addressing other significant health challenges. The meticulous research and application of CBD products, especially in studies on mental health disorders and quality of life in glioma patients, have afforded us a fresh perspective on the therapeutic potential of this treatment.

Some trials which scrutinize the role of CBD in addressing other significant health challenges (such as anxiety, pain, and quality of life) are underway, including (see Table 1 for more details): a clinical trial (NCT05753007); the GRASS study (EUCTR2020-004294-48-NL).

Through these investigations, we have clearly seen researchers striving to unravel the potential of CBD in treating glioma, particularly in alleviating symptoms such as anxiety and pain. These studies provide significant clues, pointing to CBD as a potential adjunctive treatment option that could be used alongside traditional treatment plans. As these trials progress and their results are published, we look forward to a broader understanding and application scope of CBD.

**Table 1:** Clinical trials of CBD for glioma treatment

Start/report year	Design	Drug/treatment	Population	Outcome measures	Status	References or index numbers
2013	Part A: Phase Ib, Non-randomized, Single-arm, Open-label Part B: Phase Ib, Randomized, Double-blind, Placebo-controlled	Part A: Sativex^®^; Part B: Sativex^®^ + TMZ/Placebo + TMZ	Patients with confirmed grade IV GBM after radiotherapy and first-line chemotherapy with TMZ and whose tumors showed first progression per RANO criteria (Part A n = 6; Part B n = 21)	Primary endpoint: Evaluate adverse events of Sativex^®^ alone or in combination with TMZ; Secondary endpoints: 1. Number of patients achieving PFS6; 2. Number of patients alive at the end of the trial achieving a survival period of 1 year	Completed	[6]/NCT01812603 /NCT01812616 (Absent specific designation, NCT uniformly refers to registrations found on https://clinicaltrials.gov, the same applies henceforth.)
2018	–	Pharmaceutical grade CBD oil	Patients with solid tumors ranging from prostate, breast, esophageal to gliomas, including 119 patients (glioma patients, n = 10)	The author observed the clinical response of patients taking CBD under specific protocols, including counting circulating tumor cells and imaging assessments; side effects of CBD	Completed	[73]
2023	Phase Ib, Open-label, Multicenter, Intrapatient Dose Escalation	TN-TC11G (THC:CBD = 1:1) + Radiotherapy + TMZ	Newly diagnosed GBM patients	Primary endpoint: Maximum tolerated dose of TN-TC11G; adverse event occurrence rate during treatment (National Cancer Institute-Common Terminology Criteria for Adverse Events (NCI-CTCAE) grade). Secondary endpoints: Anti-tumor effect of TN-TC11G combined with TMZ and radiotherapy; OS; PFS; Expression of midkine in peripheral blood	Recruiting Patients	NCT03529448
2018	Phase I, Single-arm	Cannabis (high CBD and low THC concentration) + Concurrent Radiochemotherapy	Patients receiving concurrent radiochemotherapy for GBM	Primary endpoint: Incidence of adverse events during treatment; Secondary endpoints: Number of radiation-induced toxic events and quantity of opioids used by patients during treatment	Ended	NCT03246113
2023	Phase II, Multicenter, Double-blind, Placebo-controlled, Randomized	Sativex^®^ + TMZ/Placebo + TMZ	Patients with MGMT promoter methylated recurrent GBM	Primary endpoint: OS. Secondary endpoints: OS at 6, 12, and 24 months; PFS; Quality of life scores assessed by the European Organisation for Research and Treatment of Cancer's (EORTC) QLQ-C30; Adverse events during treatment evaluated by NCI-CTCAE v5.0	Recruiting Patients	NCT05629702
2018	Phase I/II, Randomized, Double-blind, Placebo-controlled, Parallel, Multicenter	CBD	Patients with multiple myeloma, GBM, and gastrointestinal malignancies	Primary endpoint: response rate. Secondary endpoints: time to progression (TTP); PFS; patient-reported outcomes (PRO) Quality of Life (QoL); clinician-reported outcomes (ClinRO) QoL	Unknown	NCT03607643
2015	Phase I, Non-randomized, Single-arm, Open-label	Epidiolex^®^	Epileptic patients, including those with primary brain tumors with intractable seizures (n = 3)	Primary endpoint: Number of participants with serious adverse events (increase in seizure frequency by 100% leading to Emergency Room Visit or hospital treatment); Participants with significant clinical changes in resting blood pressure or heart rate of 25% assessed by a neurologist; Participants with clinically significant laboratory test changes. Secondary endpoints: Change in seizure frequency measured by monthly total number of seizures; Change in seizure severity measured by the Chalfont Seizure Severity Scale	Completed	NCT02700412; NCT02695537; [83, 84]
2023	Phase II, Randomized, Double-blind, Placebo-controlled	High concentration CBD full spectrum industrial hemp extract	Newly diagnosed GBM patients undergoing standard treatment with radiotherapy combined with TMZ	Primary endpoint: Change from baseline in self-assessed anxiety levels using Beck Anxiety Inventory (BAI); Emotional change from baseline using the Overall Anxiety Severity and Impairment Scale (OASIS). Secondary endpoints: Changes in baseline pain (measured with Brief Pain Inventory, Visual Analog Scale, Pain Distress Scale, Pain Disability Index), sleep changes using the the Pittsburgh Sleep Quality Index (PSQI), quality of life using the EORTC QLQ-C30 and QLQ-BN20	Recruiting Patients	NCT05753007
2021	Phase II, Double-blind, Placebo-controlled, Randomized	CBD	Patients with glioma and moderate to severe anxiety	Primary endpoint: Significant improvement in S-STAI scores. Secondary endpoints: Changes in stress, depression, QoL, and HRQoL	Ongoing	EUCTR2020-004294-48-NL (Registered on www.clinicaltrialsregister.eu/)
2011	Phase III, Double-blind, Multicenter, Randomized, Placebo-controlled	Sativex^®^/Placebo (GA-0034)	Patients with chronic pain not relieved by optimized opioid therapy for advanced cancer	Primary efficacy endpoint: Percentage improvement in average daily pain NRS score (Study 1) and average change from randomization baseline to the end of treatment in average pain NRS score (Study 2). Secondary endpoints: Changes from baseline to the end of treatment in average pain score, highest pain NRS score, and NRS score causing sleep interruption	Completed	NCT01361607; NCT01424566; [90, 91]

## CBD in conjunction with drug modification and nanoparticle delivery systems for glioblastoma treatment

**Fig. 3:**
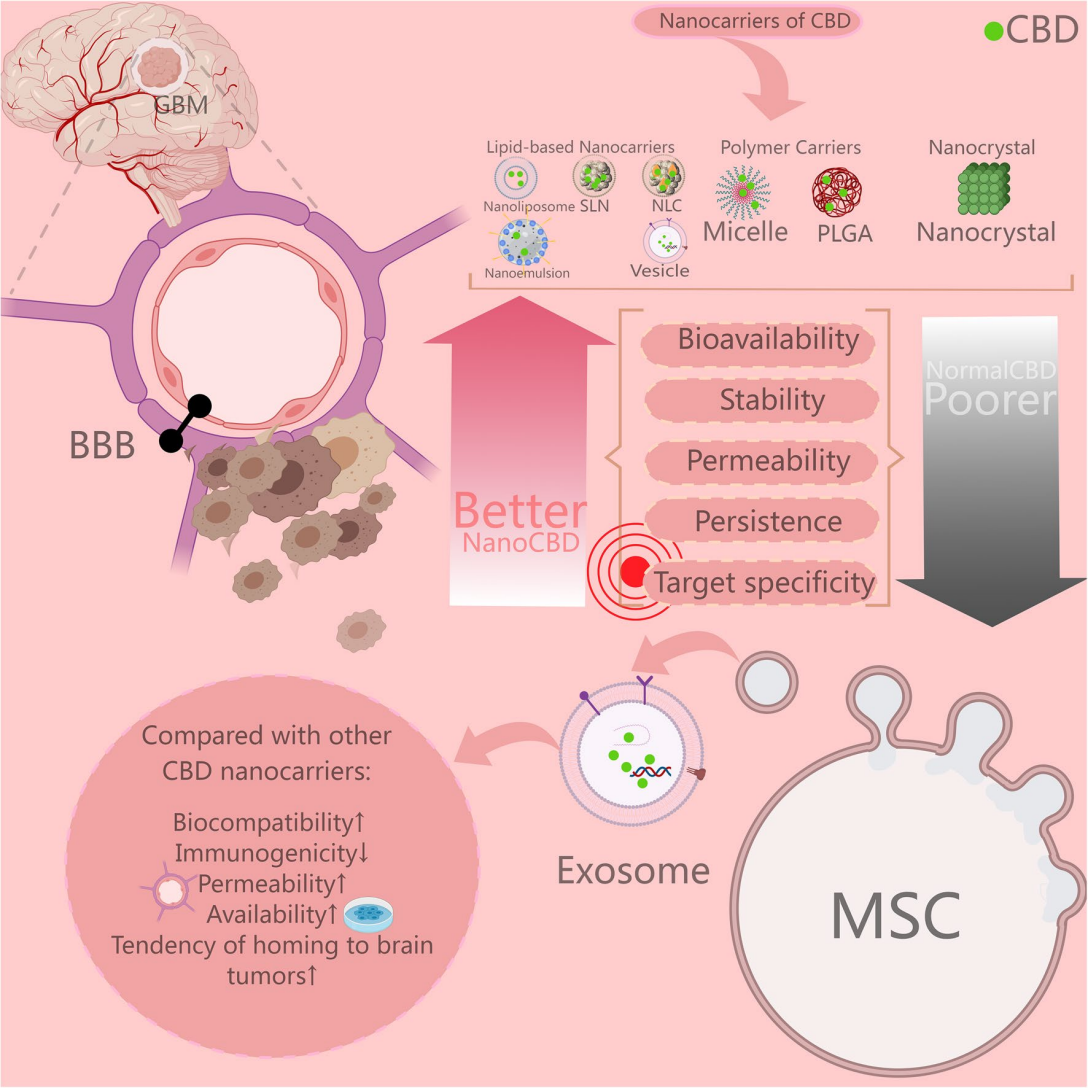
Nanoparticulate Formulations of CBD. The nanoparticulate formulations of CBD are primarily categorized into lipid nanoparticles, polymer nanocarriers, and nanocrystals. Compared to conventional CBD’s formulations, these nanoparticulate versions boost significantly enhanced solubility and stability in bodily fluids, superior biodistribution, increased bioavailability, and improved penetration through the blood–brain barrier. Notably, exosomes derived from mesenchymal stem cells, as novel drug nanocarriers, offer considerable advantages due to their ease of acquisition and preparation, exceptional biocompatibility, superior permeability through biological barriers, and enhanced active and passive targeting capabilities, making them a highly promising vehicle for drug delivery. This figure is created with MedPeer (medpeer.cn)

In the treatment of glioma, a significant challenge lies in the targeted delivery of drugs through BBB into the brain parenchyma. Many drugs fail to penetrate the BBB to any clinically meaningful extent, which is a crucial reason for the poor systemic treatment response in brain tumor patients [95]. The high lipophilicity of CBD presents challenges in drug bioavailability, distribution, and delivery: increased lipophilicity could potentially create a substrate for efflux pumps in the endothelial cells of BBB, negating the drug's entry through passive diffusion, and, similarly, heightened lipophilicity leads to reduced selectivity, augmented uptake in non-target tissues, increased tissue burden, and an expanded volume of distribution due to higher plasma protein binding rates [96]. Indeed, the nano-formulation of drugs offers enhanced bioavailability and targeting, allowing for reduced dosage and side effects, thereby decreasing the frequency of administration and increasing patient tolerance for treatment. Nanotechnology eliminates the need for chemical modification of drugs, thereby preventing functional alteration and enzymatic degradation in peripheral bodily fluids. Nanomedicines can enhance target site specificity through both passive and active targeting, such as the unique enhanced permeability and retention (EPR) effect, and surface modifications of nanomedicines aid in permeating tumor tissues. Nano-formulations enhance the solubility, encapsulation efficiency, stability, bioavailability, and sustained release of CBD. Nanotechnological assemblies of CBD are primarily categorized into lipid-based nanocarriers (such as nanoliposomes, nanoemulsions, nanostructured lipid carriers (NLCs), and solid lipid nanoparticles (SLNs), vesicles [97], etc.), polymer carriers (including micelles [98], poly-lactic-co-glycolic acid (PLGA) nanoparticles [99], etc.), and nanocrystals (as shown in Fig. 3).

A study encapsulated CBD within magnesium-gallate metal–organic framework microparticles (CBD/Mg-gallate-MOF), the majority of which were smaller than 2 μm. At a neutral pH (pH 7.2), 65% of the CBD was released, capable of inhibiting the viability of C6 glioma cells and inducing caspase-mediated apoptosis. An in vitro BBB model demonstrated that drug treatment resulted in a 53.1% reduction in transendothelial electrical resistance (TEER), highlighting its potential to penetrate the BBB [100]. The work of Aparicio-Blanco and colleagues underscores the pioneering application of lipid nanocapsules (LNCs) as targeted and sustained release carriers for CBD in the field of glioma therapy. These CBD-functionalized LNCs showed superior control over the glioma cell line U-373MG, with a targeting capability that was 3.4 times higher than that of unmodified LNCs. Notably, the anti-tumor efficacy of CBD was inversely proportional to the size of the LNCs; reducing LNCs to 20 nm significantly enhanced the release of CBD and its inhibitory effect on tumor cell growth, with the IC_50_ value decreased to one-third of that of 50 nm-sized LNCs. This innovative work not only highlights the importance of nanotechnology in optimizing drug distribution and efficacy but also offers a new perspective on the application of liquid nanocapsules in tumor therapy drug delivery systems [101, 102]. Joanna and colleagues developed a PLGA-based nanoparticle encapsulating etoricoxib and CBD using emulsification and solvent evaporation methods, treating two different human glioblastoma multiforme (GBM) cell lines (T98G and U-138 MG). Their findings indicate that the nanoparticles could reduce cell viability in a dose-dependent manner and induce programmed cell death in both GBM cell lines, presenting a potential new avenue for GBM treatment [103]. Similar efforts by Hernán Pérez de la Ossa D and others, using polycaprolactone/polyvinyl alcohol (PCL/PVA) as carriers for CBD nanoparticles, showed sustained release capabilities. In a mouse GBM xenograft model, smaller doses and fewer administration frequencies achieved the same anti-tumor growth effect as CBD dissolved in solution, while also enhancing tumor cell apoptosis and inhibiting cell proliferation and angiogenesis [48]. Zhou and colleagues developed a unique nano-drug delivery structure, Nano-reshaper, by encapsulating CBD with the lymphocyte recruitment cytokine LIGHT in lipid-calcium phosphate, effectively overcoming the short half-life of cytokines, significant side effects, and BBB permeability issues. This nanocarrier not only improved the solubility and bioavailability of the drug in water, reducing potential toxicity, but also enhanced the therapeutic effect. The research also demonstrated a significant increase in systemic T-cell numbers and local tumor T-cell infiltration in in situ GBM model mice. Furthermore, when combined with the αPD-1 immune checkpoint inhibitor, this therapeutic strategy achieved an 83.3% long-term survival rate in mice, with no recurrence observed [36]. Sun and colleagues made a substantial innovation by utilizing corn alcohol soluble protein Zein as a carrier, formulating a nanoparticle encapsulating Gboxin and CBD, GZCX. GZCX successfully enhanced BBB permeation and achieved effective targeting of GBM, leading to apoptosis of tumor cells and inhibition of angiogenesis [104]. These findings further emphasize the potential and importance of nanotechnology in treating challenging brain tumors.

Nanoparticulate formulations have enhanced the bioavailability of CBD, yet they each bear inherent limitations, such as issues with stability, cost-effectiveness, complexity of production processes, or potential safety concerns. Exosomes from mesenchymal stem cells (MSCs), which are nanoscale vesicles released by stem cells, have garnered attention in recent years as an innovative drug delivery system due to their biological properties. Utilizing MSC-derived exosomes to encapsulate drugs as a nanoparticulate formulation offers several potential advantages over traditional nanoparticle-based approaches, including improved biocompatibility and reduced immunogenicity, enhanced targeting capabilities, and an increased ability to traverse biological barriers such as the BBB [105, 106]. The efficacy of exosomes loaded with chemotherapeutic agents like doxorubicin in combating gliomas has been documented [106]. Future research could focus on the role of exosome-encapsulated CBD, a novel drug combination, in the treatment of gliomas.

## Issues should be considered in the application of CBD

Despite the promising potential of CBD in the treatment of glioma, it is imperative to thoroughly understand its potential drawbacks and unknown aspects to ensure its safe and effective application.

### Drug interactions

CBD inhibits the cytochrome P450 enzyme system (CYP450), particularly the CYP3A4 and CYP2C19 enzymes, which play a crucial role in drug metabolism [107]. Consequently, CBD may alter the plasma concentrations of other drugs metabolized by these enzymes, as previously discussed in Sect.  3.2.1. For instance, antiepileptic drugs like clobazam and antidepressants such as fluoxetine depend on the CYP450 system for metabolism. Co-administration with CBD may lead to increased drug concentrations, thereby enhancing therapeutic effects or elevating the risk of side effects [108, 109]. Additionally, certain anti-glioma medications, such as etoposide and irinotecan, are metabolized by CYP3A4, and CBD may impact their metabolic clearance, thereby affecting their anticancer efficacy [110, 111].

Furthermore, P-glycoprotein (P-gp) is a significant drug efflux transporter present in tissues such as the intestines, liver, kidneys, and the blood–brain barrier. It functions by expelling drugs from cells, thereby influencing their absorption, distribution, and excretion. CBD has been shown to inhibit P-glycoprotein activity, potentially increasing the intracellular concentrations of certain anti-glioma drugs like temozolomide, and vincristine [112–114].

### Side effects and adverse events

CBD is generally regarded as relatively safe. In a Phase III clinical trial aimed at reducing seizures in Dravet syndrome, dosages ranging from 600 mg/day to 3000 mg/day demonstrated good tolerability; in a Phase I clinical trial, dosages even reached up to 6000 mg/day [115]. Nevertheless, the potential side effects and adverse reactions of CBD warrant attention.

In animal models, reported adverse reactions include developmental toxicity, hepatocellular injury, and reproductive system damage; and in humans, common side effects encompass fatigue, diarrhea, changes in appetite, drowsiness, and vomiting [116]. At higher dosages, CBD may induce more severe side effects, such as elevated liver enzyme levels. Particularly for patients with impaired liver function, CBD might further burden the liver, necessitating caution in this patient population [117].

Moreover, CBD may cause hypotension and dizziness, which in certain instances could lead to falls and injuries [118]. CBD might also affect immune system function, although its exact mechanisms and clinical significance remain not entirely clear. This poses a potential risk for cancer patients with already compromised immune function. Therefore, when considering CBD for therapeutic use, both physicians and patients should fully account for these potential side effects and risks.

### Potential for abuse

While CBD does not exhibit the pronounced psychoactive properties of THC, its potential for abuse still warrants attention. Globally, there remains ambiguity regarding whether this substance should be regulated [119]. Current research indicates that the addiction risk associated with CBD is exceedingly low [120]. In fact, CBD may even mitigate cannabis addiction and help restore normal brain function in addicts [121, 122]. However, the variability in the quality and purity of CBD products available on the market may increase the risk of abuse [123].

### Quality control and standardization issues

Presently, numerous countries permit the purchase of various CBD products through over-the-counter (OTC) channels or online platforms, including CBD oils, capsules and tinctures for systemic use and topical ointments [119]. However, the quality of CBD products on the market is inconsistent, lacking uniform standards and regulation. Some products may contain unlisted THC or other impurities, which could not only affect efficacy but also pose legal and health risks [123]. Legally, despite the 2014 U.S. Agricultural Act distinguishing industrial hemp (defined as Cannabis sativa L. and any part of such plant, whether growing or not, with a delta-9-THC content of no more than 0.3% on a dry weight basis, while the EU sets this threshold at less than 0.2%) from marijuana, the interstate trade of CBD-containing food and dietary supplements remains illegal; furthermore, in certain states, the sale of CBD products and hemp oil is also prohibited [123]. Nonetheless, industry and regulatory trends increasingly favor the use of high-purity cannabidiol for medical purposes. Variations in production processes and extraction methods, as well as differences in the parts of the plant used, can result in significant discrepancies in CBD content, complicating precise dosage control. For clinical applications, standardized CBD formulations and stringent quality control are paramount to ensuring efficacy and safety.

Moreover, the optimal dosage and administration route of CBD in the treatment of gliomas have not been established. The dosage range commonly reported for CBD in patients with other diseases is < 1–50 mg/kg/day; and Epidiolex^®^ (which contains CBD at a concentration of 100 mg/mL), approved by the Food and Drug Administration (FDA) in 2018 for the treatment of Dravet syndrome and Lennox-Gastaut syndrome, is typically administered at a dosage of approximately 20 mg/kg/day [115]. Additionally, the potential for long-term, high-dose use to cause tolerance or dependence, as well as its impact on overall patient survival and quality of life, still requires further investigation. Therefore, long-term follow-up studies and large-scale clinical trials are crucial for assessing these long-term effects.

## Concluding and future perspectives

CBD, a non-psychoactive cannabinoid derived from the cannabis plant, has shown promising potential in the treatment of gliomas. Characterized by its safety, good tolerability, and absence of psychoactive effects, CBD induces apoptosis in glioma cells, mitochondrial dysfunction, and autophagy, thereby inhibiting the proliferation and invasion of glioma cells, suppressing the expression of GSCs properties, and promoting cell death. Additionally, it enhances the sensitivity to radiotherapy and chemotherapy while protecting neural functions, playing a significant role in the management of glioma symptoms. Preclinical and clinical studies have demonstrated encouraging anti-glioma activity. However, laboratory studies face certain limitations, such as the inability of in vitro experiments, conducted under idealized conditions with a single cell type, to fully reflect the complex microenvironment of tumors in vivo. The heterogeneity among different glioma cell lines may lead to variable research outcomes, and differences in pharmacokinetics and pharmacodynamics across species limit the extrapolation of animal model data. Moreover, in clinical settings, CBD faces challenges and limitations, such as small sample sizes and potential selection bias in current studies on its use in glioma treatment. Future research requires the design of rigorous large-scale, multicenter, randomized controlled trials to provide a more robust evidence base for research and clinical applications. Legal and regulatory obstacles also limit its lawful use in many countries and regions. Dosage form selection, dose determination, and standardization pose additional challenges that necessitate further research to identify optimal treatment doses and regimens. Additionally, patient acceptance and education regarding CBD need to be enhanced, while remaining vigilant about potential adverse reactions and drug interactions. Overall, CBD displays potential therapeutic prospects in glioma treatment. Nonetheless, further scientific research is needed to support its clinical application and address related challenges and limitations. It is hoped that future efforts will facilitate CBD's emergence as an effective adjunctive medication in glioma therapy, offering patients a wider array of treatment options.

## Data Availability

Data sharing is not applicable to this article as no datasets were generated or analysed during the current study.

## Abbreviations

AEA: Anandamide
ATF4: Activating transcription factor 4
BAI: Beck anxiety inventory
BBB: Blood brain barrier
BMC: Biomolecular condensate
CBD: Cannabidiol
ClinRO: Clinician-reported outcome
CNS: Central nervous system
CSSS: Chalfont seizure severity scale
CXCL-16: C-X-C motif chemokine 16
CYP450: Cytochrome P450 enzyme system
DDIT3: DNA damage inducible transcript 3
DIT: Dose-intense temozolomide
DNA: Deoxyribonucleic acid
EORTC: European organisation for research and treatment of cancer
EPR: Enhanced permeability and retention
ER: Endoplasmic reticulum
FAAH: Fatty acid amide hydrolase
FDA: Food and drug administration
GBM: Glioblastoma multiforme
GSC: Glioma stem cell
HIF-1α: Hypoxia-inducible factor-1α
HSP: Heat shock protein
IDO: Indoleamine 2,3-Dioxygenase
JNK1/2: C-Jun N-terminal Kinase 1 and 2
LOX: Lipoxygenase
LNC: Lipid Nanocapsule
MAPK: Mitogen-activated protein kinase
MCP-1: Monocyte chemotactic protein-1
MGMT: O6-Methylguanine-DNA Methyltransferase
MMP-9: Matrix Metalloproteinase-9
MSC: Mesenchymal stem cell
MTOR: Mammalian target of rapamycin
NCI-CTCAE: National cancer institute-common terminology criteria for adverse events
NLC: Nanostructured lipid carrier
NRS: Numeric rating scale
NSCs: Neural stem cells
OASIS: Overall anxiety severity and impairment scale
OS: Overall survival
OTC: Over-the-counter
PCL/PVA: Polycaprolactone/polyvinyl alcohol
PDGF-AA: Platelet-derived growth factor-AA
PFS6: 6-Month progression-free survival
PI3K: Phosphoinositide 3-Kinase
PKC ζ: Protein Kinase C ζ
PLGA: Poly-lactic-co-glycolic acid
P-gp: P-glycoprotein
PRO: Patient-reported outcome
PSQI: Pittsburgh sleep quality index
PTEN: Phosphatase and tensin homolog
QoL: Quality of life
RELA: V-rel reticuloendotheliosis viral oncogene homolog A
RNA: Ribonucleic acid
ROS: Reactive oxygen species
RR: Response rate
Ser-PAI-1: SerpinE1-plasminogen activator inhibitor type-1
SGs: Stress granules
SLN: Solid lipid nanoparticle
STAT3: Signal transducer and activator of transcription 3
TGF-β1: Transforming growth factor-β1
THC: Tetrahydrocannabinol
TIMP1/4: Tissue inhibitors of metalloproteinase 1 and 4
TMZ: Temozolomide
TRIB3: Tribbles Pseudokinase 3
TRPV2: Transient receptor potential Vanilloid 2
TRPV4: Transient receptor potential Vanilloid 4
TTP: Time to progression
uPA: Urokinase plasminogen activator
VDAC1: Voltage-dependent anion channel 1
VEGF: Vascular endothelial growth factor
WHO: World Health Organization
